# Altered Serum MicroRNA Profile May Serve as an Auxiliary Tool for Discriminating Aggressive Thyroid Carcinoma from Nonaggressive Thyroid Cancer and Benign Thyroid Nodules

**DOI:** 10.1155/2019/3717683

**Published:** 2019-09-16

**Authors:** Aisen Zhang, Cheng Wang, Hui Lu, Xi Chen, Yi Ba, Chunni Zhang, Chen-Yu Zhang

**Affiliations:** ^1^State Key Laboratory of Pharmaceutical Biotechnology, Nanjing Advanced Institute for Life Sciences, School of Life Sciences, Jiangsu Engineering Research Center for MicroRNA Biology and Biotechnology, Nanjing University, Nanjing, China; ^2^Department of Gerontology, The First Affiliated Hospital with Nanjing Medical University, Nanjing, China; ^3^Department of Clinical Laboratory, Jinling Hospital, Nanjing University School of Medicine, Nanjing University, Nanjing, China; ^4^Department of General Surgery, The First Hospital Affiliated to Nanjing Medical University, Nanjing, China; ^5^Tianjin Medical University Cancer Institute and Hospital, Key Laboratory of Cancer Prevention and Therapy, Tianjin, China

## Abstract

Thyroid cancers are the most common malignancy of the endocrine system; however, there is no reliable blood biomarkers for thyroid cancer diagnosis and even for aggressive and nonaggressive thyroid cancers as well as benign nodule discrimination. The present study is aimed at evaluating whether circulating microRNA (miRNA) can differentiate aggressive and nonaggressive thyroid cancer from benign thyroid nodules. In this study, we performed a multiphase, case-control study to screen serum miRNA expression profile in 100 patients with papillary thyroid cancer (PTC), 15 patients with aggressive medullary thyroid carcinoma (MTC), 91 patients with benign nodules, and 89 healthy controls using TaqMan low-density array followed by extensive reverse transcription quantitative real-time PCR validation. The results showed that the serum levels of miR-222-3p, miR-17-5p, and miR-451a were markedly increased, while miR-146a-5p, miR-132-3p, and miR-183-3p were significantly decreased in the PTC and benign nodule groups compared with the control group. There was no difference in the miRNA expression profile between the PTC group and the benign nodule group. Nevertheless, the serum levels of miR-222-3p and miR-17-5p were significantly increased in the MTC group than the benign nodule and control group. Moreover, receiver operating characteristic curve analyses demonstrated that the 2 miRNAs and their panel can accurately discriminate MTC from the benign nodule group and healthy controls. These findings indicated that the altered circulating miRNAs may discriminate PTC and benign thyroid nodules from controls, and serum miR-222-3p and miR-17-5p have the potential to serve as auxiliary tools for diagnosing more aggressive thyroid carcinomas, such as MTC.

## 1. Introduction

Thyroid cancers are the most common malignancy in the endocrine system and comprise 1% of all cancers. Differentiated thyroid carcinomas (DTCs), which include papillary and follicular cancers, comprise the vast majority (90%) of total thyroid cancer cases [1]. In the past three decades, even with the progress made on diagnostic methods, the yearly incidence of thyroid cancer has increased nearly 3-fold in the United States and continues to rise [2]. The increase has been attributed to the higher incidence of papillary thyroid carcinomas (PTCs), especially small PTCs (2 cm or smaller) [1], which comprises over 80% of all cancer cases. Thyroid nodules have a prevalence of 4-50% based on the diagnostic methods and patients' age. Most thyroid nodules are benign; only 5% are malignant [3], but suspicious thyroid nodules must be tested for malignancy. Currently, thyroid ultrasound and the fine-needle aspiration (FNA) are the two most valuable diagnostic approaches. The ultrasound examination is noninvasive, but heavily dependent on the techniques and clinical experience of the operators. FNA is now the gold standard procedure for the differential diagnosis of thyroid nodules. However, there are still limitations, such as inadequate sampling and indeterminate results (10-40%) [4]. In these situations, repeat aspirations or diagnostic lobectomy are recommended [5], although more than 50% of the patients who underwent diagnostic lobectomy were finally found to be benign [4]. In addition, the FNA diagnostic procedure is invasive. All these reasons bring difficulties in differential diagnosis and result in patient suffering. Blood-based examination is ideal for clinical diagnosis as it is minimally invasive. Currently, no clinically routine blood test is available for thyroid cancer. Therefore, the identification of noninvasive biomarkers is urgently needed for the differentiation of aggressive thyroid carcinoma from nonaggressive thyroid cancer and benign thyroid nodules.

MicroRNAs (miRNAs) are a class of endogenous, small, noncoding RNAs of 19–23 nt cleaved from larger hairpin precursors. By binding to the 3′-untranslated region of target mRNA, miRNAs can cause a block of translation or mRNA degradation. They comprise one of the most abundant classes of gene regulatory molecules in multicellular organisms and take part in many biological processes, including proliferation, apoptosis, differentiation, and carcinogenesis [6]. Numerous studies have noted that expression of particular miRNAs is found in various human cancer tissues, including thyroid cancer [7–13], and several miRNAs (miR-221, miR-222, miR-146b, miR-181b, miR-155, miR-183, and miR-223) were found to be upregulated in postoperative cancer tissues, formalin-fixed paraffin-embedded tissues, and FNA samples. Furthermore, researches indicated that these dysregulated miRNAs are helpful in distinguishing cancer from normal or benign nodule tissues [14–16]; however, the invasiveness of the procedures has limited its application.

Our group and others have previously discovered that miRNAs are stably present at sufficient levels in circulating blood to be detected as blood-based biomarkers [17, 18]. Subsequent, researchers have identified distinct expression signatures of circulating miRNA in a variety of cancers, such as lung cancer, hepatocellular and renal carcinoma, esophagus cancer, and pancreatic cancer [19–23]. To date, a few studies have focused on the profile of circulating miRNA in thyroid tumors [24–29]. However, although differential miRNA expressions in thyroid carcinomas (most PTCs) have been reported, the results are highly inconsistent. In our present study, we screened the serum miRNA expression profile by high-throughput TaqMan low-density array (TLDA) followed by extensive reverse transcription quantitative real-time PCR (RT-qPCR) validation. We compared the serum miRNA profiles of PTC patients with that of benign thyroid nodules and healthy controls. In addition, we recruited 15 patients with more aggressive medullary thyroid carcinomas (MTC) for miRNA detection.

## 2. Materials and Methods

### 2.1. Patients and Control Subjects

A total of 295 participants including 100 patients with PTC, 91 patients with benign nodules, 15 patients with MTC, and 89 healthy controls were enrolled in the study. Patients with primary PTC or benign thyroid nodules were recruited from the First Affiliated Hospital of Nanjing Medical University (Nanjing, China), between Feb 20, 2012, and Sep 8, 2012. Patients with MTC were recruited from Tianjin Medical University Cancer Institute and Hospital (Tianjin, China), between Feb 20, 2013, and Nov 8, 2013. The 89 healthy control subjects were age- and gender-matched volunteers with no current or previous malignancy and no thyroid disease. The surgical procedure was performed on all patients, and final diagnoses were based upon pathological examination. The relevant demographic and clinical pathological information of patients with histologically confirmed PTC, MTC, or benign nodules was recorded. Written informed consent was obtained from all patients and healthy volunteers prior to the study. This study was approved by the ethics committees of the above participating institutions in accordance with the Declaration of Helsinki.

### 2.2. Blood Collection and RNA Isolation

Venous blood (approximately 5 mL) was collected from each patient before surgery, as well as from healthy controls. The blood was centrifuged at 1,500 g for 10 min at room temperature. The supernatant (serum) was separated from the cellular layer, transferred to a fresh tube, and stored at -80°C until further analysis. For the TLDA analysis, equal volumes of serum from each patient (500 *μ*L) were pooled in each group (20 PTC patients, 20 benign nodule patients, and 20 healthy controls, 10 mL serum from each group). TRIzol reagent (Invitrogen, Carlsbad, CA) was used to extract total RNA from each pool of serum samples as previously described [17]. For the RT-qPCR assay, total RNA was extracted from 100 *μ*L serum with a 1-step phenol/chloroform purification protocol as previously described [19].

### 2.3. Analysis of Serum miRNAs by TDLA

For the TLDA analysis, reverse transcription was performed using the TaqMan MicroRNA Reverse Transcription Kit and Megaplex RT Primers as previously described [17]. Briefly, 3 *μ*L total RNA was reverse transcribed to cDNA in RT reaction mix. A preamplification was performed after the reverse transcription to generate enough cDNA templates. Then, the TLDA was performed on a profiling of 756 different human miRNAs. The miRNA expression was presented as CT values and normalized to an internal control (*Δ*CT) according to the manufacturer's recommendation. The fold change of each miRNA was determined as 2^−*ΔΔ*CT^.

### 2.4. Analysis of Serum miRNAs by RT-qPCR

A TaqMan probe-based qRT-PCR assay was performed according to the manufacturer's instructions (Applied Biosystems, Foster City, CA, USA), with minor modifications as described previously [17]. Briefly, 2 *μ*L of RNA extracted from serum was reverse transcribed to cDNA using reverse transcriptase (TaKaRa, Dalian, China) and a stem-loop RT primer (Applied Biosystems, Foster City, CA, USA). Real-time PCR was then performed on the Applied Biosystems 7500 Sequence Detection System with TaqMan probe (Applied Biosystems, Foster City, CA, USA). The final CT value of each miRNA was determined using a fixed threshold setting. Our previous study has indicated that a combination of let-7d, let-7g, and let-7i shows highly stability across normal controls and patients with diseases and is statistically superior to the most commonly used reference genes in the quantification of serum miRNAs [30–32]. Therefore, in this study, let-7d/g/i was used as an endogenous control for normalizing the data of experimental qRT-PCR for serum miRNAs. Relative levels of miRNA were normalized to the let-7d/g/i and were calculated as 2^−*Δ*CT^. *Δ*CT was then calculated by subtracting the CT values of let-7d/g/i from the average CT values of the miRNAs of interest.

### 2.5. Statistical Analysis

All statistical analyses were performed using the Statistical Analysis System software SPSS 19.0. Data were presented as the mean ± SEM for miRNAs or mean ± S.D. for other variables. Statistical significance of the difference in the demographic features between groups was determined using Student's *t*-test or two-sided *χ*^2^ test. Student's *t*-test was used to determine the significance of different miRNA expression levels. All *P* values were two-sided, and a *P* value <0.05 was considered to be statistically significant. A risk score analysis was performed to evaluate the associations between serum miRNA levels and PTC. In brief, the risk score of each miRNA, denoted as *s*, was set to 1 if its concentration was higher than the upper 95% reference interval (for the upregulated miRNA in PTC group) or than the lower 5% reference interval (for the downregulated miRNA in PTC group) for the corresponding miRNA concentration in controls and to 0 otherwise. A risk score function to predict PTC was defined according to a linear combination of concentration for each miRNA. For example, the RSF for sample *i* using information from the altered miRNAs was RSF_*i*_ = ∑^*n*^_*j*−1_*W*_*j*_ · *s*_*ij*_. In the above equation, *s*_*ij*_ is the risk score for miRNA *j* on sample *i*, and *W*_*j*_ is the weight of the risk score of miRNA *j*. To determine the *W*_*s*_, the univariate logistic regression models were fitted using the disease status with each of the risk scores. The regression coefficient of each risk score was used as the weight to show the contribution of each miRNA to the RSF. We constructed the ROC curves and calculated the area under the ROC curves to evaluate the predictive power of candidate miRNAs for PTC and MTC.

## 3. Results

### 3.1. Patient Characteristics

**Figure 1:**
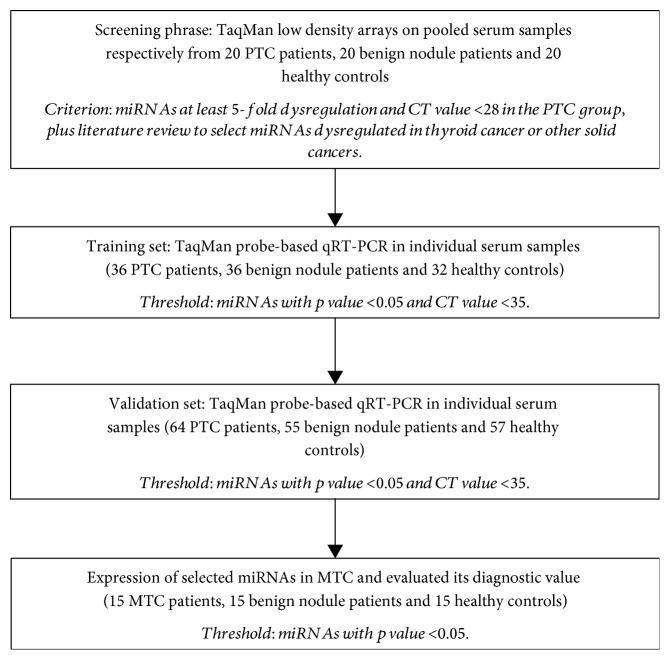
Overview of the design strategy.

A multiphase, case-control study was designed to identify serum miRNAs as surrogate biomarkers for PTC (Figure 1).  Table 1 shows the demographic and clinical features of the patients with PTC or benign nodules and healthy controls. There was no significant difference in the distribution of age or gender between PTC patients, benign nodule patients, or healthy controls.

### 3.2. Screening of Candidate Serum miRNAs in PTC, Benign Nodule, and Healthy Control Groups

In the initial biomarker screening stage, we employed a TLDA technique to screen the expression levels of 756 miRNAs in pooled serum samples from patients with PTC, patients with benign nodules, and healthy controls (each pooled from 20 individuals). We searched for potential miRNA signatures to distinguish patients with PTC from those with benign nodules and healthy controls fulfilled all the following criteria: (1) threshold cycle (CT) value < 28 cycles in the PTC group; (2) at least 5-fold change in expression in the PTC group compared with the benign group or the healthy control group; and (3) literature review miRNAs dysregulated in thyroid cancer or other solid cancers. As a consequence, we established a list of 31 differentially expressed miRNAs for further analysis (see [Supplementary-material supplementary-material-1] in the Supplementary Material for comprehensive data analysis).

### 3.3. Confirmation of miRNA Level by RT-qPCR Analysis

In the training phase, the 31 miRNAs chosen from TLDA were measured in a separate set of individual serum samples from 36 patients with PTC, 36 patients with benign nodules, and 32 healthy controls. Only miRNAs with a *P* value <0.05 were selected for further analysis. Two miRNAs, miR-151-3p and miR-19b-3p, were excluded from further examination, because their Cq values in most of samples were larger than 35. The expression levels of miR-222-3p, miR-17-5p, and miR-451a were markedly increased, while miR-146a-5p, miR-132-3p, and miR-183-3p were significantly decreased in the PTC group and the benign nodules group when compared with the healthy control group; however, no significant difference in the levels of these 6 miRNAs was found between the PTC group and the benign nodules group (see [Supplementary-material supplementary-material-1] in the Supplementary Material for comprehensive data analysis). The other 23 miRNAs were not significantly changed among the three groups.

**Figure 2:**
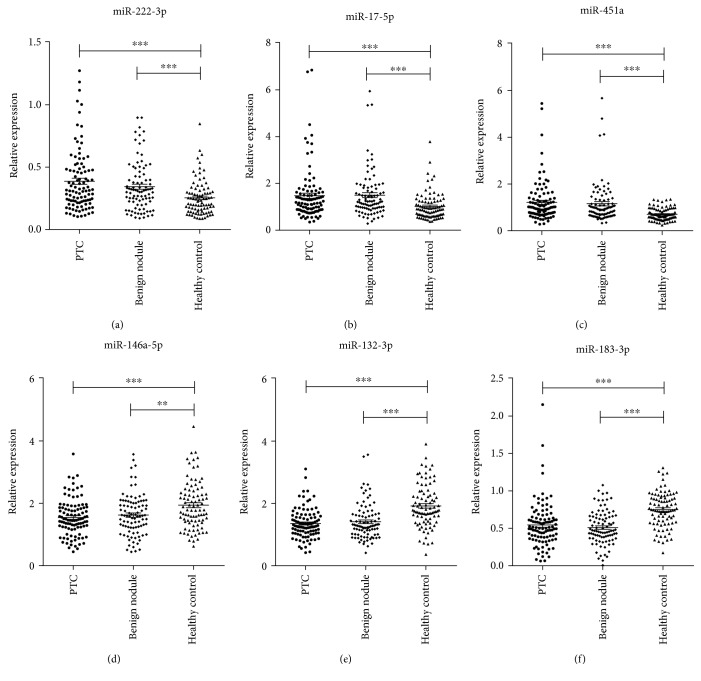
The 6 serum miRNAs' signatures in the PTC group or the benign nodule group vs. the healthy control group. Relative expression levels of 6 miRNAs were measured in 100 PTC patients, 91 benign nodule patients, and 89 healthy control individuals with a TaqMan probe-based qRT-PCR assay. CT values were normalized to let-7d/g/i, and the relative expression was shown as 2^-*Δ*CT^. ^∗∗^*P* < 0.01, ^∗∗∗^*P* < 0.001.

The above 6 miRNAs were further examined in an additional validation set consisting of 64 PTC patients, 55 matched benign nodule patients, and 57 matched healthy controls. Consistent with the results from the training set, miR-222-3p, miR-17-5p, and miR-451a were significantly elevated, while miR-146a-5p, miR-132-3p, and miR-183-3p were reduced in the PTC and the benign nodule groups compared with the control group (miR-146a-5p expression was not statistically different between the benign nodule group and the healthy control group). Still no significant differences in these miRNAs were found between the PTC group and the benign group (Table 2).  Figure 2 shows the differences in the concentration of the 6 miRNAs in the total 100 PTC patients, 91 benign nodule patients, and 89 healthy controls enrolled in the training and validation sets.

We subsequently evaluated the performance of the six serum miRNAs as well as their panel in the training and validation sets for discriminating PTC from controls. As can be seen in [Supplementary-material supplementary-material-1], ROC curve analysis yielded the AUC of the six individual miRNAs that are ranging from 0.653 (95% CI 0.575-0.732) to 0.765 (95% CI 0.696-0.835) for discriminating PTC from healthy controls. To further test the diagnostic ability of the combination of the six miRNAs, we performed a risk score analysis on the data set and ROC curve analysis was then used to evaluate the diagnostic effect of the six-miRNA panel. As shown in [Supplementary-material supplementary-material-1], the AUC for the combination of the six miRNAs was 0.772 (95% CI 0.704-0.839), which is obviously higher than any miRNA alone. Thus, based on the above results, we believe that the combination of the six miRNAs is a more robust indicator of PTC than any miRNA alone.

### 3.4. Expression Level of miRNAs in MTC

**Figure 3:**
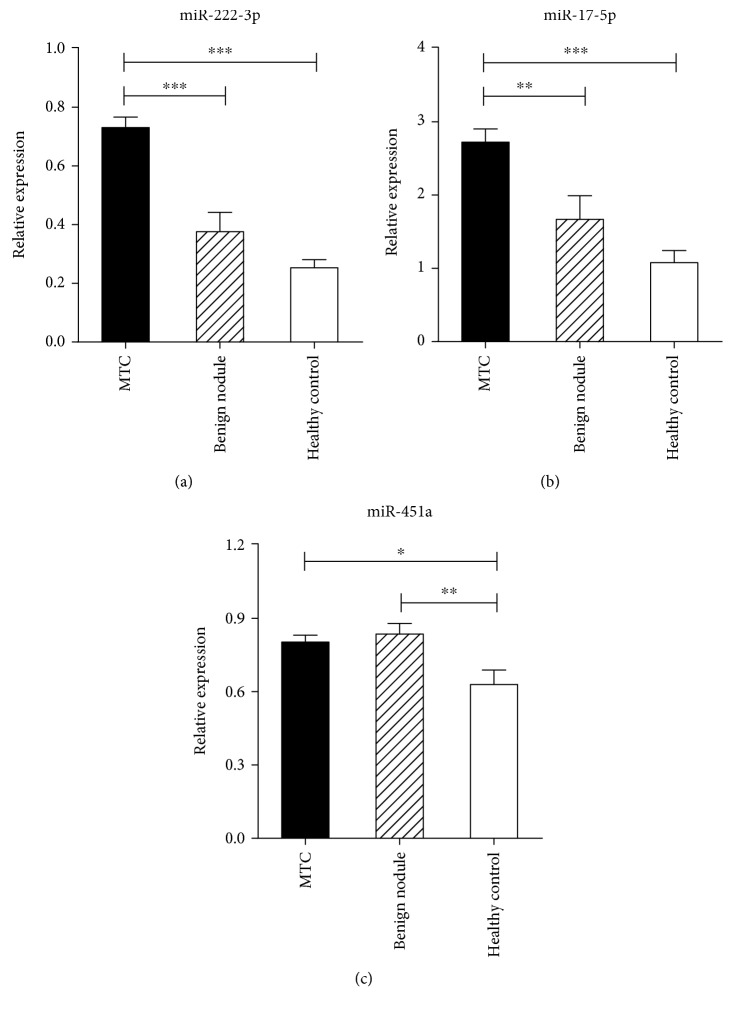
The profile of 3 serum miRNAs in MTC. Relative expression levels of 3 miRNAs were measured in 15 MTC patients, 15 benign nodule patients, and 15 healthy control individuals with a TaqMan probe-based qRT-PCR assay. CT values were normalized to let-7d/g/i, and the relative expression was shown as 2^-*Δ*CT^. ^∗^*P* < 0.05, ^∗∗^*P* < 0.01, ^∗∗∗^*P* < 0.001.

**Figure 4:**
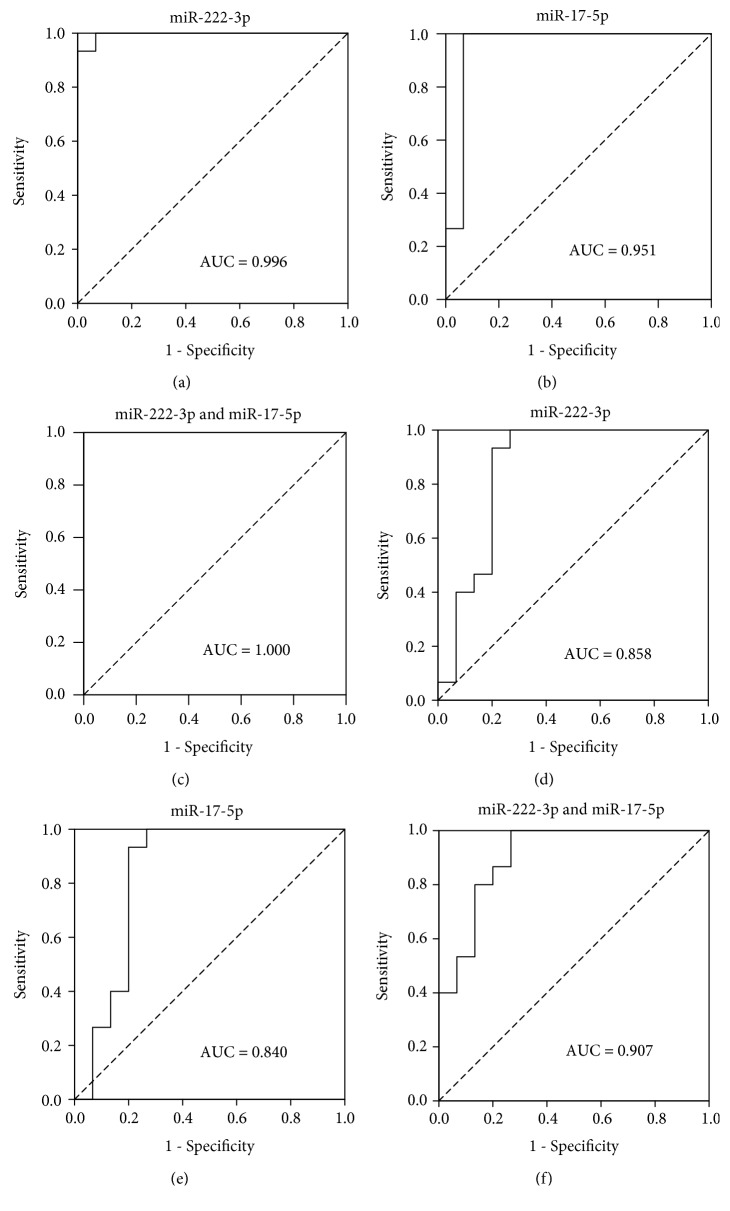
The ROC curves of 2 serum miRNAs in the MTC patients compared with healthy controls or the benign nodule subjects. ROC curves indicate the ability of serum analysis of the 2 individual miRNAs, miR-222-3p and miR-17-5p, in individuals or combined, to differentiate cases of MTC from healthy controls (a–c) and the benign nodule subjects (d–f).

As we know, PTC is relatively indolent and has a favorable prognosis with an 85% 10-year survival [33]. Many PTCs never resulted in any clinical symptoms during patients' lives. To some extent, PTC behaves much like a benign nodule, which may explain the lack of difference in the serum miRNA expression between PTC and benign nodule patients. Unlike PTC, MTC, which was originated from parafollicular C cells, is considered more aggressive than PTC. We selected and examined the above 3 upregulated miRNAs (miR-222-3p, miR-17-5p, and miR-451a) in patients with MTC (Supplementary [Supplementary-material supplementary-material-1]) and found that the expression profile of these miRNAs in MTC patients was significantly different from that in benign nodule patients. The levels of the 2 miRNAs, miR-222-3p and miR-17-5p, were markedly higher in MTC patients than in age- and sex-matched benign nodule patients and healthy controls (Table 3 and Figure 3). We performed receiver operating characteristic (ROC) curve analyses on the 2 miRNAs and obtained the respective area under the ROC curves (AUCs). For MTC and healthy control group, AUCs are 0.996 (95% CI 0.981-1.000) and 0.951 (95% CI 0.856-1.000) for miR-222-3p and miR-17-5p, respectively; for the MTC and benign nodule group, AUCs are 0.858 (95% CI 0.707-1.000) and 0.840 (95% CI 0.674-1.000). We further evaluate the diagnostic value of the two-miRNA profiling system and find the AUC of combined miR-222-3p and miR-17-5p is 1.000 (95% CI 1.000-1.000) for MTC patients from healthy control patients and 0.907 (95% CI 0.799-1.000) for discriminating MTC from the benign nodule group (Figure 4). The above data indicate that the 2 serum miRNAs (miR-222-3p and miR-17-5p) can discriminate MTC from benign nodules or healthy controls with high diagnostic accuracy.

## 4. Discussion

Numerous studies have demonstrated the association between the aberrant miRNA expression and the development of thyroid cancer; however, these studies mainly focused on miRNAs expressed in tumor tissues and FNA samples [9, 10, 13, 15, 34]. Given that this procedure is invasive, it is inappropriate for thyroid nodule screening. Studies by our group and other laboratories have demonstrated that numerous stable miRNAs are expressed in circulating blood and can be readily detected by various assays such as Solexa sequencing technology, miRNA microarray, and RT-qPCR. More importantly, the distinctive miRNA expression profiles in patients with various cancers, including lung, colorectal, prostate, and pancreatic cancers, may serve as biomarkers for their detection. A serum miRNA-based biomarker is advantageous for analyzing tumors without the need of biopsy, surgery, or other invasive procedures. The sources of circulating miRNA remain unclear; however, our previous study and others have demonstrated that circulating miRNAs were derived not only from tissues affected by disease but also from cancer-related immune responses; thus, circulating miRNAs may carry more comprehensive information about the diagnosis and prognosis of the disease [17].

Until now, there have been several studies on circulating miRNA expression in PTC patients. Yu et al. found the expression levels of serum miR-222, miR-151-5p, and let-7e were elevated in PTC patients when compared with benign nodule patients and healthy controls [24]. In Lee et al.'s research, the main focus was on whether plasma miRNAs could be a biomarker for PTC recurrence, but did not address their use for differential diagnosis of thyroid nodules, and the author found that circulating miRNAs (miR-222 and miR-146b) did not seem to be able to distinguish between malignant and benign follicular growth [25]. In a Caucasian population, Cantara et al. found that miR-95 was downregulated, and miR-190 was upregulated in PTC patients' sera compared with nodule goiters and healthy subjects [26]. Graham et al.'s study in 31 Canadians revealed that serum miR-146a-5p and miR-199b-3p were downregulated, while let-7b-5p and miR-10a-5p were upregulated between PTC and benign nodule samples, but the significant differences between benign and PTC patients disappeared after adjusted for multiple comparators [27]. In Korea, Lee et al. selected 4 miRNAs, including miR-146b, miR-222, miR-221, and miR-155, and examined their plasma levels in 89 patients (70 PTCs and 19 benign lesions). MiR-146b and miR-155 were higher in the PTC group than the benign group, but with relatively lower AUC values (0.649-0.695) [28]. Recently, another research in Turkey analyzed seven miRNAs in sera of patients with PTC, multinodular goiter (MG), and healthy controls. In the preoperative miRNA expression, only miR-21 was significantly upregulated in PTC and MG groups compared with the control group; no differences were found between the PTC and MG groups, and none of the possible combinations of miRNAs achieved sufficient discriminative power to distinguishing PTC from the benign disease [29]. The results reported in these literatures are highly inconsistent.

To verify whether circulating miRNA profile in papillary thyroid cancer can discriminate from benign thyroid nodules, in this study, we used the next generation of high-throughput TLDA combined with RT-qPCR to compare the expression levels of a circulating miRNA profile in serum samples of PTC patients, benign nodule patients, and matched healthy individuals. We selected as many as 31 miRNAs observed to be dysregulated in PTC patients from pooled serum using TLDA analysis and then confirmed the expression levels of these candidate miRNAs with a RT-qPCR assay of samples from 100 PTC patients, 91 benign nodule patients, and 89 healthy controls in the training and validation sets. We found that the expression levels of miR-222-3p, miR-17-5p, and miR-451a were significantly upregulated, whereas miR-146a-5p, miR-132-3p, and miR-183-3p were downregulated in the PTC and the benign nodule groups when compared with the healthy control group, even though no difference was found in all these miRNAs between the PTC group and the benign nodule group. Furthermore, we also noticed that the dysregulated serum miRNAs in PTC patients which identified in our present study are less overlap with those previous studies. We suspected that these inconsistences may be generated by the difference in the method and the normalization approach used between our study and those previous studies, for instance, the different internal control (let-7d/g/i) and the pooled serum samples for initial miRNA screening were used in our present study.

PTCs are the most common histological subtype of thyroid cancers, being approximately 80% of all cases. In the past three decades, PTC diagnoses increased by approximately 3- to 4-fold, contributing to the principle increase of total thyroid cancers, whereas the incidences of other types, including follicular thyroid carcinoma (FTC), anaplastic thyroid carcinoma (ATC), and MTC, remained stable. Most of this increase (87%) is attributed to tumors that are 2 cm or smaller [2], which occurs at a similar incidence in our study (82%). In clinical practices, PTCs are the least aggressive histological type among all thyroid carcinomas and are sometimes called an indolent cancer as revealed by the following evidence: (1) PTCs are common in mankind and have a favorable prognosis with an 85% 10-year survival; most do not influence the patients' lives. Tumors, 2 cm or smaller, have an even better prognosis [33, 35]. (2) Although the incidence of PTC has been increasing in many countries over the last 30 years, the mortality has been slowly decreasing [2]. (3) Research in Japan in which patients with small PTCs had been followed for up to 10 years had favorable results, less than 10% of the tumors grew to 3 mm or more, and 23% of these tumors decreased in size in the following observation [36]. Since the beginning of 2014, a number of articles noted the overdiagnosis in thyroid cancer cases, and overtreatment may be of limited benefit [37–40]. And the latest ATA practice guidelines also pay attention to the problem of overdiagnosis and overtreatment of DTCs which is typically indolent, and recommend more surveillance management and less aggressive treatment (total thyroidectomy and radioactive iodine) of thyroid nodules and DTCs, especially PTCs [41]. Together, the above evidences demonstrate that PTCs, especially small ones, may have similar biological behavior and prognosis as benign nodules. That may be the reason why we cannot find any difference of miRNA expression levels between PTC and benign nodules. And on the other hand, our and others' results also provide some evidences that PTC in general is not more aggressive than nodule.

MTC, originated from parafollicular C cells, which is different from PTC, is considered to be more aggressive [42]. In this study, we also recruited 15 MTC patients just for example of more malignant thyroid carcinomas. We found that two serum miRNAs, namely, miR-222-3p and miR-17-5p, were significantly upregulated compared with benign nodule patients and healthy controls. ROC curves of the two miRNAs in individual or combined showed that they could distinguish MTC from benign nodules or healthy controls with high accuracy. However, further studies are necessary in serially acquired blood samples of a larger cohort of patients.

Expression patterns of miR-222 and miR-17 in tissues and cells have been investigated in thyroid cancers. Studies indicated that miR-222 was upregulated in tissue and FNA cell samples [33]. This miRNA, together with miR-221, targets p27^*kip*1^, p57, PTEN, TIMP3, and c-KIT, and it may play an essential role in thyroid carcinogenesis [43]. MiR-17-5p, a member of miR-17-92a cluster, is upregulated in thyroid cancers and several other types of cancers and plays an important role in several pathological ways. Takakura et al. reported that the miR-17-92 cluster, containing seven miRNAs (miR-17-5p, miR-17-3p, miR-18a, miR-19a, miR-20a, miR-19b, and miR-92-1), was overexpressed in ATC cell lines. MiR-17-5p was also overexpressed in human ATC samples compared to normal tissue [44].

A critical problem in research on circulating miRNAs is proper normalization for accurate miRNA quantification. Several methods, such as normalizing miRNA concentration to serum volume, applying external miRNAs, or using endogenous miRNAs or small RNAs, are commonly used. Applying external miRNAs might not be a good choice, because external controls cannot reflect the variability in serum sampling. In addition, small RNAs such as U6, RNU6B, RNU44, and RNU48 are not stable enough in serum or plasma, and their concentrations vary between samples. We also found that miRNA like miR-16, which has been used as an internal reference in some circulating miRNA research, was not an appropriate normalizing factor [30, 45]. In our previous study, we demonstrated that a combination of let-7d, let-7g, and let-7i (let-7d/g/i) can be a suitable reference for the normalization of serum miRNAs, and it is statistically superior to some existing methods [30–32]. Thus, we used let-7d/g/i as an endogenous control for normalization of serum miRNA levels to guarantee reliability of our results.

There were some disadvantages in this study, such as a lack of risk stratification analysis, a small number of MTC patients, and a lack of FTC or ATC samples. Future studies will focus on expanding the research to include these parameters.

## 5. Conclusions

In conclusion, our present study showed that the profile of 6 altered serum miRNAs (miR-222-3p, miR-17-5p, miR-451a, miR-146a-5p, miR-132-3p, and miR-183-3p) might represent a new potential tool for discriminate PTC and benign thyroid nodules from controls, and patients with least aggressive PTC displayed a similar serum miRNA profile as patients with benign nodules. In addition, two serum miRNAs, including miR-222-3p and miR-17-5p, have the potential to serve as biomarkers for differential diagnosis of MTC from benign thyroid nodules. In future, more effects are needed in studying circulating miRNAs in more aggressive PTCs or other thyroid carcinomas.

## Figures and Tables

**Table 1:** Demographic and clinical features of PTC patients, benign nodule patients, and healthy control individuals in the training and validation sets.

Variables	PTC (*n* = 100)	Benign nodule (*n* = 91)	Healthy control (*n* = 89)	*P* value^b^	*P* value^c^
Age (years)^a^	44.8 ± 14.2	45.4 ± 12.5	44.9 ± 13.4	0.744^d^	0.941^d^
Age group, *n* (%)					
<45 years	50 (50%)	41 (45%)	45 (51%)	0.562^e^	1^e^
≥45 years	50 (50%)	50 (55%)	44 (49%)		
Sex, *n* (%)					
Male	23 (23%)	18 (20%)	18 (20%)	0.602^e^	0.725^e^
Female	77 (77%)	73 (80%)	71 (80%)		
Tumor size, *n* (%)					
≤2 cm	82 (82%)				
>2 cm	18 (18%)				
LN metastasis, *n* (%)					
Yes	38 (38%)				
No	62 (62%)				
Multifocal tumor, *n* (%)					
Yes	40 (40%)				
No	60 (60%)				
TNM stage, *n* (%)					
I/II	77 (77%)				
III/IV	23 (23%)				

PTC: papillary thyroid cancer. ^a^Age data are presented as the mean ± S.D.^b^Difference between the PTC group and the benign nodule group. ^c^Difference between the PTC group and the healthy control group. ^d^Student's *t*-test. ^e^Two-sided *χ*^2^ test.

**Table 2:** MiRNA levels in the PTC, benign nodule, and the healthy control groups in the validation set by qRT-PCR^a^.

miRNA	PTC (*n* = 64)	Benign nodule (*n* = 55)	Healthy control (*n* = 57)	*P* value^b^	*P* value^c^	*P* value^d^
miR-222-3p	0.415 ± 0.030	0.390 ± 0.026	0.290 ± 0.020	0.536	<0.001	<0.01
miR-17-5p	1.590 ± 0.139	1.771 ± 0.159	1.152 ± 0.086	0.391	<0.05	<0.001
miR-451a	0.964 ± 0.060	0.862 ± 0.045	0.639 ± 0.030	0.188	<0.001	<0.001
miR-146a-5p	1.627 ± 0.082	1.816 ± 0.094	2.065 ± 0.113	0.128	<0.01	0.092
miR-132-3p	1.442 ± 0.067	1.566 ± 0.086	2.052 ± 0.104	0.247	<0.001	<0.001
miR-183-3p	0.527 ± 0.042	0.500 ± 0.033	0.733 ± 0.035	0.619	<0.001	<0.001

PTC: papillary thyroid cancer. ^a^The relative contents of miRNAs are normalized to let-7d/g/i and presented as the mean ± SEM. ^b^Difference between the PTC group and the benign nodule group. ^c^Difference between the PTC group and the healthy control group. ^d^Difference between the benign nodule group and the healthy control group.

**Table 3:** Differentially expressed miRNAs among the MTC, benign nodule, and the healthy control groups by qRT-PCR^a^.

miRNA	MTC (*n* = 15)	Benign nodule (*n* = 15)	Healthy control (*n* = 15)	*P* value^b^	*P* value^c^	*P* value^d^
miR-222-3p	0.731 ± 0.035	0.376 ± 0.066	0.253 ± 0.028	<0.001	<0.001	0.098
miR-17-5p	2.719 ± 0.184	1.669 ± 0.322	1.079 ± 0.163	<0.01	<0.001	0.113
miR-451a	0.803 ± 0.028	0.836 ± 0.043	0.629 ± 0.059	0.533	<0.05	<0.01

MTC: medullary thyroid carcinoma. ^a^The relative contents of miRNAs are normalized to let-7d/g/i and presented as mean ± SEM. ^b^Difference between the MTC group and the benign nodule group. ^c^Difference between the MTC group and the healthy control group. ^d^Difference between the benign nodule group and the healthy control group.

## Data Availability

No data were used to support this study.

## Supplementary Materials

Supplementary Materials Table S1: dysregulated miRNAs in pooled serum samples from the PTC, benign nodule, and the healthy control groups determined by TLDA. Table S2: differentially expressed miRNAs among the PTC, benign nodule, and the healthy control groups in the training set by qRT-PCR1. Table S3: the AUCs of the 6 serum miRNAs and their panel in ROC curves analysis for PTC and controls. Table S4: demographic and clinical features of the MTC patients, matched benign nodule patients, and healthy control individuals.
