# Supplementary material for: Altered Serum MicroRNA Profile May Serve as an Auxiliary Tool for Discriminating Aggressive Thyroid Carcinoma from Nonaggressive Thyroid Cancer and Benign Thyroid Nodules
Source: Dis Markers. 2019 Sep 16;2019:3717683. doi: 10.1155/2019/3717683 (PMC6766139; doi:10.1155/2019/3717683)
Supplement: Supplementary Materials — Table S1: dysregulated miRNAs in pooled serum samples from the PTC, benign nodule, and the healthy control groups determined by TLDA. Table S2: differentially expressed miRNAs among the PTC, benign nodule, and the healthy control groups in the training set by qRT-PCR1. Table S3: the AUCs of the 6 serum miRNAs and their panel in ROC curves analysis for PTC and controls. Table S4: demographic and clinical features of the MTC patients, matched benign nodule patients, and healthy control individuals. [file 3717683.f1.pdf]

Supplementary Materials

Table S1 ｜Dysregulated miRNAs in pooled serum samples from the PTC, benign nodule and the healthy control groups determined by TLDA.

miRNA	PTC (ΔCT) [a]	Benign nodule (ΔCT) [a]	Healthy control (ΔCT) [a]	Fold change [b] (PTC vs Healthy controls)	Fold change [c] (PTC vs Benign nodule)
miR-140-5p	5.7458230	11.96381	21.488104	54814.91957	74.439012
miR-374b-5p	7.7001343	21.555685	21.488104	14144.65825	14823.00882
miR-15b-5p	7.9054585	21.555685	21.488104	12268.28103	12856.64417
miR-664-3p	8.853712	21.685385	21.89232	8414.18603	7289.84791
miR-22-3p	8.714579	21.555685	21.488104	7001.87213	7337.66843
miR-28-5p	9.310087	21.555685	21.488104	4633.91864	4856.15246
miR-148a-3p	9.566719	21.555685	21.488104	3878.77399	4064.79252
miR-132-3p	6.2873898	10.835986	17.29107	2053.23095	23.40259
miR-93-5p	3.0036297	6.2375793	11.464775	352.41837	9.40840
miR-19b-3p	0.9770184	7.188507	8.98852	258.04907	74.10447
miR-19a-3p	7.697296	14.396744	15.490163	221.76179	103.92853
miR-26a-5p	7.285074	12.263776	14.78407	180.89340	31.53107
miR-20b-5p	4.847439	8.976614	12.280873	172.85685	17.49869
miR-223-5p	5.6076183	11.732357	12.981915	165.91456	69.77986
miR-451a	2.055975	6.1466846	9.41103	163.71640	17.03830
miR-199a-3p	7.461588	13.192363	14.675967	148.50616	53.10497
miR-17-5p	0.4355450	3.3007717	7.5077343	134.56779	7.28650
miR-30a-3p	5.4614906	13.609775	12.348399	118.34939	283.71220
miR-24-3p	-0.0267582	4.0160027	6.7012825	106.00883	16.48133
miR-628-3p	8.611599	21.685385	15.133647	91.90351	8621.87541
miR-151a-5p	8.314804	14.14999	14.826596	91.25249	57.09079
miR-126-3p	1.3874226	4.389454	7.3859043	63.93268	8.01127
miR-146b-5p	3.2094193	6.5567627	8.940588	53.11946	10.17773
miR-26b-3p	7.5953484	12.498836	13.26855	51.02745	29.92932
miR-195-5p	5.048443	11.53008	10.681157	49.61533	89.36494
miR-183-3p	9.590357	21.685385	14.904515	39.78515	4374.88082
miR-186-5p	1.8360672	5.710184	6.81583	31.55426	14.66309
miR-200c-3p	9.367243	16.817154	14.290509	30.34246	174.84237
miR-16-5p	-1.3614216	4.176815	3.241455	24.29987	46.47029
miR-146a-5p	-1.0971565	1.3083973	3.4137611	22.79930	5.29839
miR-222-3p	-0.9773254	1.6525078	3.1795883	17.83839	6.18954

[a] $\Delta CT = CT^{miRNA} - CT^{internal\ control}$

[b] Fold change=$2^{-[\Delta CT(PTC\ miRNA)-\Delta CT(healthy\ control\ miRNA)]}$

[c] Fold change=$2^{-[\Delta CT(PTC\ miRNA)-\Delta CT(henign\ nodule\ miRNA)]}$

Table S2 | Differentially expressed miRNAs among the PTC, benign nodule and the healthy control groups in the training set by qRT-PCR[1]

**miRNA**	**PTC n=36**	**Benign nodule n=36**	**Healthy control n=32**	***p*-value[2]**	***p*-value[3]**	***p*-value[4]**
miR-222-3p	0.3364 ± 0.0436	0.2709 ± 0.0301	0.1889 ± 0.0155	**0.2207**	0.0034**	0.0225*
miR-17-5p	1.2620 ± 0.1858	1.0810 ± 0.0554	0.7718 ± 0.0480	**0.3537**	0.0182*	0.0001***
miR-451a	1.6690 ± 0.1950	1.6080 ± 0.2007	0.8249 ± 0.0493	**0.8293**	0.0002***	0.0006***
miR-140-5p	0.3620 ± 0.0320	0.3463 ± 0.0341	0.3436 ± 0.0289	**0.7387**	0.6740	0.9516
miR-374b-5p	0.1878 ± 0.0071	0.1926 ± 0.0036	0.2008 ± 0.0044	**0.5566**	0.1522	0.1553
miR-15b-5p	0.0491 ± 0.0041	0.0508 ± 0.0043	0.0492 ± 0.0026	**0.7757**	0.9751	0.7603
miR-664-3p	5.0540 ± 0.2458	5.0470 ± 0.2550	4.615 ± 0.1709	**0.9845**	0.1437	0.1594
miR-22-3p	0.1188 ± 0.0086	0.1131 ± 0.0060	0.1278 ± 0.0059	**0.5854**	0.3971	0.0858
miR-28-5p	0.0181 ± 0.0010	0.0179 ± 0.0011	0.0175 ± 0.0006	**0.8518**	0.5875	0.7584
miR-148a-3p	0.1362 ± 0.0080	0.1403 ± 0.0104	0.1590 ± 0.0084	**0.7587**	0.0558	0.1669
miR-132-3p	1.2020 ± 0.0461	1.1750 ± 0.0572	1.6880 ± 0.0645	**0.7146**	0.0001***	0.0001***
miR-93-5p	0.3828 ± 0.0218	0.3998 ± 0.0261	0.3465 ± 0.0192	**0.6192**	0.2209	0.1107
miR-19a-3p	0.0815 ± 0.0033	0.0800 ± 0.0031	0.0807 ± 0.0022	**0.7475**	0.8332	0.8661
miR-26a-5p	1.0340 ± 0.0984	1.1240 ± 0.1448	0.8233 ± 0.0525	**0.6114**	0.0723	0.0672
miR-20b-5p	0.3311 ± 0.0139	0.3330 ± 0.0165	0.3343 ± 0.0134	**0.9313**	0.8721	0.9531
miR-223-5p	0.1619 ± 0.0077	0.1557 ± 0.0071	0.1566 ± 0.0072	**0.5537**	0.6140	0.9285
miR-199a-3p	0.1887 ± 0.0125	0.1933 ± 0.0141	0.1945 ± 0.0102	**0.8058**	0.7178	0.9453
miR-30a-3p	0.5273 ± 0.0559	0.4944 ± 0.0292	0.4933 ± 0.0269	**0.6087**	0.5859	0.9777
miR-24-3p	0.3141 ± 0.0191	0.2997 ± 0.0176	0.3190 ± 0.0213	**0.5816**	0.8632	0.4828
miR-628-3p	0.1418 ± 0.0183	0.1305 ± 0.0180	0.1744 ± 0.0262	**0.6595**	0.3136	0.1732
miR-126-3p	1.0890 ± 0.0928	1.1160 ± 0.1493	0.8840 ± 0.0928	**0.8909**	0.1880	0.1930
miR-146b-5p	2.6900 ± 0.1887	2.4720 ± 0.1303	2.7170 ± 0.1286	**0.3449**	0.9088	0.1872
miR-26b-3p	0.4883 ± 0.0237	0.4882 ± 0.0239	0.4451 ± 0.0193	**0.9985**	0.1620	0.1658
miR-195-5p	0.1894 ± 0.0106	0.1884 ± 0.0087	0.1804 ± 0.0083	**0.9440**	0.5162	0.5125
miR-183-3p	0.5777 ± 0.0393	0.5242 ± 0.0304	0.7800 ± 0.0299	**0.2856**	0.0002***	0.0001***
miR-186-5p	0.8651 ± 0.0487	0.9168 ± 0.0344	0.9060 ± 0.0241	**0.3904**	0.4553	0.7984
miR-200c-3p	0.0260 ± 0.0019	0.0251 ± 0.0018	0.0244 ± 0.0012	**0.7317**	0.4668	0.7253
miR-16-5p	0.0845 ± 0.0048	0.0785 ± 0.0067	0.0727 ± 0.0041	**0.4671**	0.0674	0.4671
miR-146a-5p	1.3920 ± 0.0664	1.3190 ± 0.0738	1.7110 ± 0.0890	**0.4633**	0.0049**	0.0011**
miR-151a-5p					Ct > 35	
miR-19b-3p					Ct > 35	

[1]The relative contents of miRNAs are presented as mean±SEM.

[2] Difference between the PTC group and the benign nodule group.

[3] Difference between the PTC group and healthy control group.

[4] Difference between the benign nodule group and healthy control group.

[5] * $p<0.05$, ** $p<0.01$, *** $p<0.001$.

Table S3 | The AUCs of the 6 serum miRNAs and their panel in ROC curves analysis for PTC and controls.

miRNA	AUC	Std. Errora	Asymptotic 95% Confidence Interval	
			Lower Bound	Upper Bound
**miR-222-3p**	0.680	0.038	0.604	0.755
**miR-17**	0.665	0.039	0.588	0.742
**miR-451a**	0.757	0.035	0.688	0.825
**miR-146a**	0.653	0.04	0.575	0.732
**miR-132**	0.765	0.035	0.696	0.835
**miR-183**	0.752	0.036	0.681	0.822
**miR-Panel**	0.772	0.035	0.704	0.839

Table S4 | Demographic and clinical features of the MTC patients, matched benign nodule patients and healthy control individuals.

Variables	MTC n=15	Benign nodule n=15	Healthy control n=15	p^b	p^c
**Age, years**[a]	50.5(10.1)	50.4(9.8)	50.1(10.3)	0.9855[d]	0.9292[d]
**Sex, No.**					
Male	7(47%)	7(47%)	7(47%)	1[e]	1[e]
Female	8(53%)	8(53%)	8(53%)		
**LN metastasis, n**					
Yes	13(87%)				
No	2(13%)				
**TNM stage, n**					
I/II	13(87%)				
III/IV	2(13%)				

[a] Age data are presented as the mean (SD).

[b] Difference between PTC group and benign nodule group.

[c] Difference between PTC group and healthy control group.

[d] Student's *t*-test.

[e] Two-sided χ^2 test.
